# First Total Synthesis of a Naturally Occurring Iodinated 5′-Deoxyxylofuranosyl Marine Nucleoside

**DOI:** 10.3390/md10040881

**Published:** 2012-04-10

**Authors:** Jianyun Sun, Yanhui Dou, Haixin Ding, Ruchun Yang, Qi Sun, Qiang Xiao

**Affiliations:** Jiangxi Key Laboratory of Organic Chemistry, Jiangxi Science Technology Normal University, Nanchang 330013, China; Email: sjy089@163.com (J.S.); tdlybh521@163.com (Y.D.); dinghaixin@yahoo.cn (H.D.); ouyangruchun@yahoo.cn (R.Y.); dr.purevision@gmail.com (Q.S.)

**Keywords:** Vorbrüggen glycosylation, total synthesis, pyrrolo[2,3-*d*]pyrimidine, marine nucleoside

## Abstract

4-Amino-7-(5′-deoxy-β-D-xylofuranosyl)-5-iodo-pyrrolo[2,3-*d*]pyrimidine **1**, an unusual naturally occurring marine nucleoside isolated from an ascidan, *Diplosoma* sp., was synthesized from D-xylose in seven steps with 28% overall yield on 10 g scale. The key step was Vorbrüggen glycosylation of 5-iodo-pyrrolo[2,3-*d*]pyrimidine with 5-deoxy-1,2-*O*-diacetyl-3-*O*-benzoyl-D-xylofuranose. Its absolute configuration was confirmed.

## 1. Introduction

Naturally occurring deazapurine nucleosides, such as tubercidin [1], toyocamycin [2], sangivamycin [3], cadeguomycin [4] and recently isolated marine nucleoside 5-iodo-5′-deoxytubercidin, mycalesine **A** and **B** [5], showed significant biological activities. Structurally related pyrrolo[2,3-*d*]pyrimidine nucleosides have triggered the continued interest of medicinal chemists in the past decades [6,7,8].

Although many uncommon nucleosides have been isolated from terrestrial and marine organisms [5,9,10], nucleosides containing D-xylofuranose are rare in nature [11]. In 2008, 4-amino-7-(5′-deoxy-β-D-xylofuranosyl)-5-iodo-pyrrolo[2,3-*d*]pyrimidine **1** was first isolated by Japanese scientists from an ascidan, *Diplosoma* sp. It was found that nucleoside **1** causes complete inhibition of cell division in fertilized sea urchin eggs at 1 µg/mL concentration [12]. This compound is a potential lead for development of new insecticides. As part of our continuing effort for synthesis of bioactive natural 7-deazapurine nucleosides, we report the first total synthesis and structure confirmation of **1**.

## 2. Results and Discussion

In nucleoside chemistry, Vorbrüggen glycosylation (Silyl-Hilbert-Johnson reaction) is one of the most efficient approaches for nucleoside synthesis. This reaction has been widely applied in academic and industrial research [13,14,15]. According to the proposed mechanism of Vorbrüggen glycosylation, silylated nucleobase attacks the intermediate oxonium to give desired nucleoside. Different from purine and pyrimidine nucleobases, such as adenine and thymine, pyrrolo[2,3-*d*]pyrimidines (7-deazapurine) were seldom used as donors in Vorbrüggen glycosylation. The reason might be possibly ascribed to the nonreactive nature of N-7 in pyrrolo[2,3-*d*]pyrimidines [16,17].

**Figure 1:**
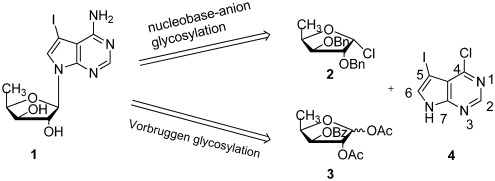
Chemical structure of pyrrolo[2,3-*d*]pyrimidine nucleoside **1** and its retrosynthetic analysis.

In order to circumvent this problem, nucleobase-anion glycosylation protocol was developed for the synthesis of 7-deazapurine nucleosides and their analogues by Robins [18,19] and Seela [20,21,22]. In this approach, labile α-D-furanosyl chloride intermediates, such as **2**, must be prepared via a lengthy synthetic route with poor yield [23,24] (Figure 1). This drawback limits its application. 

**Scheme 1:**
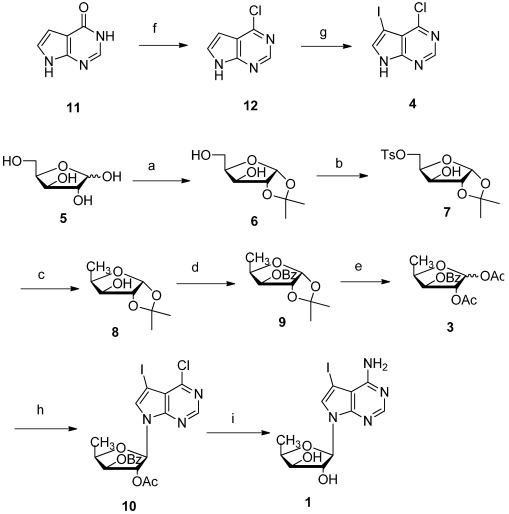
Total synthesis of pyrrolo[2,3-*d*]pyrimidine nucleoside **1**. Reagents and conditions: (**a**) (a1) Conc. H_2_SO_4_, acetone, 0 °C, 3 h; (a2) 5% Na_2_CO_3_ aq., 30 min, 87%; (**b**) TsCl (1.1 eq.), THF, Et_3_N, 0 °C, overnight, 92%; (**c**) LiAlH_4_ (0.5 eq.), THF, reflux, 6 h, 95%; (**d**) BzCl (1.1 eq), CH_2_Cl_2_, Et_3_N, 2 h, 98%; (**e**) Conc. H_2_SO_4_, Ac_2_O, 26 h, 78%; (**f**) POCl_3_ (excess), reflux, 3 h, 98%; (**g**) NIS (1.01 eq.), DMF, 0 °C, 2 h, 98%; (**h**) **4** (1 eq.), BSA (1.2 eq.), TMSOTf (1 eq.), CH_3_CN, rt-80 °C, 12 h 56%; (**i**) Sat. NH_3_ in MeOH (excess), 130 °C, 12 h, 82%.

From a synthetic point of view, Vorbrüggen glycosylation is one of the ideal choices for synthesis of pyrrolo[2,3-*d*]pyrimidine nucleosides because of commercial availability of carbohydrate acceptors. Recently, it was found that pyrrolo[2,3-*d*]pyrimidine nucleobases with electro-withdrawing groups (such as Cl, Br, I, *etc*.) at 5-position can be successfully used as donors in Vorbrüggen glycosylation for the preparation of pyrrolo[2,3-*d*]pyrimidine nucleosides with good yields [25,26,27,28]. However, its application in synthesis of xylofuranose pyrrolo[2,3-*d*]pyrimidine nucleosides has not been reported. It is important to further prove this protocol’s generality and reproducibility. We herein report a practical and efficient synthesis of **1** from D-xylose with application of Vorbrüggen glycosylation as the key step (Scheme 1).

5-Deoxy-D-xylose glycosylation acceptor **3** was synthesized starting with D-xylose **5**. Crystalline 1,2-*O*-isopropylidene-α-D-xylofuranose **6** was prepared in 87% yield by sulfuric acid-catalyzed acetalation of D-xylose, followed by partial hydrolysis with aqueous sodium carbonate added directly to the crude acetalation mixture in one pot [29]. Then 5-OH was selectively tosylated with *p*-toluenesulfonyl chloride and triethylamine in THF to afford monotosylate **7** in 92% yield. After refluxing with 2 equiv. of LiAlH_4_ in anhydrous THF, the tosylate was reduced to a methyl group in excellent 95% yield to afford **8** [30]. Subsequent benzoylation of 3-OH gave compound **9** in almost quantitative yield. Finally, the acetonide **9** was transformed to D-xylose diacetate glycosylation acceptor **3** as a mixed isomers (α:β = 2:3) on 50 g scale [31].

For preparation of nucleobase **4**, chlorination of pyrrolo[2,3-*d*]pyrimidin-4-ol **11** with phosphoryl chloride gave 4-chloro-7*H*-pyrrolo[2,3-*d*]pyrimidine **12** in 93% yield [32,33]. The reaction of **12** with 1.05 equiv. of NIS in DMF at room temperature afforded the iodinated base **4** in 95% yield on 100 g scale [25].

Next, we intended to synthesize the target molecule **1** using Vorbrüggen glycosylation. After extensive optimization of various Lewis acids, solvents, reaction temperature, it was found that the best condition were the useof 1.2 equiv. of bis(trimethylsilyl)acetamide (BSA) as silylating reagent, 2 equiv. oftrimethylsilyl triflate (TMSOTf) as Lewis acid, freshly distilled acetonitrile as solvent, and heating at 80 °C for 12 h. Then 1 equiv. 5-iodo-7*H*-pyrrolo[2,3-*d*]pyrimidine **4** was first treated with 1.2 equiv. of BSA, followed by 1.2 equiv. of 5′-deoxy-1,2,3-tri-*O*-acetyl-β-D-xylofuranose **3** and 2 equiv. of TMSOTf at room temperature. The resulting mixture was heated to 80 °C for 12 h. After workup, column chromatography afforded 4-chloro-7-(5′-deoxy-β-D-xylofuranosyl)-5-iodo-pyrrolo[2,3-*d*]pyrimidine **10** in 56% yield as pure β anomer on 20 g scale. The glycosylation yield was lower than that of the corresponding 5′-deoxy-1,2,3-tri-*O*-acetyl-D-ribofuranose acceptor, which typically gave more than 80% yield [25]. It may be due to the steric hindrance of 3′-β substitute. At last, **10** was suspended in a saturated solution of ammonia in methanol, and the resulting solution was heated at 130 °C in an autoclave for 12 h to remove the protecting ester groups and substitute the 4-Cl by 4-NH_2_on the base. The target nucleoside **1** [12] was obtained in 82% yield on 10 g scale (Scheme 1), which is sufficient for further biological studies and structure modification.

Because both D and L carbohydrate are present in marine natural products, Ueda and coworkers synthesized dibenzoates of **1** and 1-methyl-*O*-5-deoxy-β-L-xylofuranoside and compared their CD spectra to determine the absolute configuration of **1** [12]. According to their opposite cotton effect curves, the absolute configuration of **1** was indirectly determined to be D. In order to further confirm its precise configuration, the CD spectrum of synthesized **1** was determined and found identical to reported data [λ_ext_ 242 nm (∆ε −1.9) and λ_ext_ 210 nm (∆ε −2.6)]. Furthermore, because the starting material is D-xylose, the absolute configuration of nucleoside **1** is undoubtedly D. All other spectral data are in agreement with that of the reported natural nucleoside **1** [12].

## 3. Experimental Section

### 3.1. General

4-Chloro-5-iodo-7*H*-pyrrolo[2,3-*d*]pyrimidine was synthesized in our lab on 500 g scale. BSA and TMSOTf were purchased from Sigma Aldrich. MeCN was dried over CaH_2_ and distilled prior to use. Thin layer chromatography was performed using silica gel GF-254 plates (Qing-Dao Chemical Company, Qingdao, China) with detection by UV (254 nm), or charting with 10% sulfuric acid in ethanol. Column chromatography was performed on silica gel (200–300 mesh, purchased from Qing-Dao Chemical Company, Qingdao, China). NMR spectra were recorded on a Bruker AV400 spectrometer and chemical shifts (δ) are reported in ppm. ^1^H NMR and ^13^C NMR spectra were calibrated with TMS as internal standard and coupling constants (*J*) are reported in Hz. The ESI-HRMS were obtained on a Bruker Dalton microTOFQ II spectrometer in positive ion mode.

### 3.2. Synthesis of 1,2-*O*-Isopropylidene-α-D-xylofuranose *6*

D-Xylose (20.0 g, 134 mmoL) was suspended in acetone (500 mL) containing concentrated H_2_SO_4_ (20.0 mL, 98%). The mixture was stirred for 30 min at room temperature. Then a solution of Na_2_CO_3_ (26.0 g, 246 mmol) in water (224 mL) was added carefully with cooling to 0 °C. After addition, the mixture was stirred for a further 3 h. Then, solid Na_2_CO_3_ (14.0 g, 132 mmoL) was added in 3 portions over 30 min. The resulted Na_2_SO_4_ was filtered off and washed with acetone. The combined filtrates were evaporated to give crude **6**, which was purified by a silica gel column (CHCl_3_:Me_2_CO = 18:1, R_f_ = 0.32) to afford crystallized **6** (22.2 g, 87%). mp 42–43 °C; ^1^H NMR (400 MHz, CDCl_3_) δ: 5.97 (d, *J*= 4.4 Hz, 1H), 4.51 (d, *J* = 4.4 Hz, 1H), 4.31 (d, *J* = 2.0 Hz, 1H), 4.27 (d, *J* = 4.4 Hz, 1H), 4.16 (q, 1H), 4.09 (d, *J* = 4.8 Hz, 1H), 4.03 (d, *J*= 2.8 Hz, 1H), 2.04 (s, 1H), 1.47 (s, 3H), 1.31 (s, 3H); ^13^C NMR (100 MHz, CDCl_3_) δ: 111.8, 104.8, 85.6, 78.8, 78.7, 61.0, 26.7, 26.2.

### 3.3. Synthesis of 5-*O*-Tosylate-1,2-*O*-isopropylidene-α-D-xylofuranose *7*

A solution of TsCl (83.9 g, 440 mmoL) in THF (500 mL) was added slowly to 1,2-*O*-isopropylidene-α-D-xylofuranose **6** (76.1 g, 400 mmol) in THF (400 mL) and Et_3_N (300 mL) at 0 °C. After addition, the solution was stirred at room temperature overnight. CH_3_OH (10 mL) was added to quench the reaction. After filtration, the resulting solution was evaporated to dryness. The residue was dissolved in EtOAc (500 mL) and washed with H_2_O (100 mL), sat. NaHCO_3_ (100 mL) and brine (100 mL), then dried with anhydrous Na_2_SO_4_. After filtration, the solution was evaporated under reduced pressure. The residue was purified over a silica gel column (EtOAc:Hexane = 1:1) to afford **7** (126.7 g, 92%). mp 134–135 °C; ^1^H NMR (400 MHz, CDCl_3_) δ: 7.80 (d, *J* = 8.4 Hz, 2H), 7.36 (d, *J* = 8.0 Hz, 2H), 5.88 (d, *J* = 4.8 Hz, 1H), 4.52 (d, *J* = 3.6 Hz, 1H), 4.34~4.31 (m, 3H), 4.13 (q, 1H), 2.46 (s, 3H), 2.27 (d, *J *= 4.8 Hz, 1H), 1.59 (s, 1H), 1.47 (s, 3H), 1.30 (s, 3H); ^13^C NMR (100 MHz, CDCl_3_) δ: 145.3, 132.4, 130.0, 128.0, 112.2, 104.9, 85.0, 76.7, 74.3, 66.0, 26.8, 26.2, 21.7; MS (ESI) *m/z* 345.2 [M + H]^+^.

### 3.4. Synthesis of 5-Deoxy-1,2-*O*-isopropylidene-α-D-xylofuranose *8*

LiAlH_4_ (3.8 g, 100 mmol) was added in portions to 5-*O*-tosylate-1,2-*O*-isopropylidene-α-D-xylofuranose **7** (68.9 g, 200 mmol) in anhydrous THF (800 mL). The resulting suspension was refluxed for 6 h. EtOAc (50 mL) was added slowly to quench the reaction. Then H_2_O (100 mL) was added and the suspension was stirred for 2 h at room temperature. After filtration over Celite, the filtrate was evaporated to remove THF. EtOAc (500 mL) was added. The solution was washed with H_2_O (100 mL), sat. NaHCO_3_ (100 mL) and brine (100 mL), then dried with anhydrous Na_2_SO_4_. After filtration, the solution was evaporated under reduced pressure. The residue was purified over a silica gel column (EtOAc:Hexane = 1:2, R_f_ = 0.35) to afford **8** (33.0 g, 95%) as white solid. mp 81–83 °C; ^1^H NMR (400 MHz, CDCl_3_) δ: 5.83 (d, *J* = 3.6 Hz, 1H), 4.47 (d, *J* = 3.6 Hz, 1H), 4.25 (t, 1H), 3.93 (s, 1H), 2.78 (d, *J* = 4.0 Hz, 1H), 1.44 (s, 3H), 1.24 (d, *J *= 8.0 Hz, 6H); ^13^C NMR (100 MHz, CDCl_3_) δ: 111.4, 104.3, 85.5, 76.2, 76.1, 26.5, 26.1, 12.8; MS (ESI) *m/z* 175.3 [M + H]^+^.

### 3.5. Synthesis of 5-Deoxy-3-*O*-benzoyl-1,2-*O*-isopropylidene-α-D-xylofuranose *9*

Benzoyl chloride (8.71 g, 62 mmol) was added slowly to a solution of 5-deoxy-1,2-*O*-isopropylidene-α-D-xylofuranose **8** (9 g, 51.7 mmol) in anhydrous pyridine (100 mL) at 0 °C. The resulting solution was stirred for 2 h at room temperature. Ethanol (0.5 mL) was added to quench the reaction. It was evaporated under reduced pressure to dryness. The residue was dissolved in DCM (300 mL) and washed with H_2_O (50 mL), sat. NaHCO_3_ (50 mL) and brine (50 mL), then dried with anhydrous Na_2_SO_4_. After filtration, the resulting solution was evaporated under reduced pressure. The residue was purified over a silica gel column (EtOAc:Hexane = 1:3, R_f_ = 0.3) to afford **9** (14.0 g, 98%) as white solid. ^1^H NMR (400 MHz, CDCl_3_) δ: 8.02 (d, *J *= 7.6 Hz, 2H), 7.57 (t, 1H), 7.41 (t, 2H), 5.95 (d, *J* = 3.6 Hz, 1H), 5.32 (d, *J* = 2.4 Hz, 1H), 4.64 (d, *J* = 3.6 Hz, 1H), 4.54~4.49 (m, 1H),1.52 (s, 3H), 1.29 (t, 6H); ^13^C NMR (100 MHz, CDCl_3_) δ: 165.3, 133.3, 129.7, 129.4, 129.4, 128.5, 128.5, 111.6, 104.6, 83.9, 78.0, 76.9, 75.2, 26.6, 26.1, 13.3; MS (ESI) *m/z* 279.2 [M + H]^+^.

### 3.6. Synthesis of 5-Deoxy-3-*O*-benzoyl-1,2-*O*-diacetate-D-xylofuranose *3*

5-Deoxy-3-*O*-benzoyl-1,2-*O*-isopropylidene-α-D-xylofuranose **9** (7.15 g, 25.7 mmol) was dissolved in AcOH (130 mL) and Ac_2_O (150 mL). Concentrated H_2_SO_4_ (7.5 mL) was added dropwise. The solution was stirred at room temperature for 26 h. The solution was poured into H_2_O (500 mL) and extracted with DCM (3 × 100 mL). The combined organic layer was washed with H_2_O (3 × 50 mL), sat. NaHCO_3_ (3 × 50 mL) and brine (50 mL), then dried with anhydrous Na_2_SO_4_. After filtration, the resulting solution was evaporated under reduced pressure to afford **10** (6.5 g, 78%) as anomers. It was used directly without further purification. 

### 3.7. Vorbrüggen Glycosylation of 5′-Deoxy-1,2,3-*tri*-*O*-acetyl-β-D-xylofuranose 5 and 5-Iodo-7*H*-pyrrolo*[*2,3-*d]*pyrimidine *10*

To a suspension of 4-chloro-5-iodo-7*H*-pyrrolo[2,3-*d*]pyrimidine **4** (23.9 g, 85.5 mmoL) in dry MeCN (350 mL) was added BSA (20.9 g, 25 mL, 103 mmol) and stirred for 10 min at room temperature. After addition of 5-deoxy-3-*O*-benzoyl-1,2-*O*-diacetate-D-xylofuranose **3** (27.5 g, 85.5 mmol), TMSOTf (19.0 g, 15.5 mL, 85.5 mmol) was added to the mixture. The mixture was stirred for 15 min before heating to 80 °C for 12 h. The reaction was cooled to room temperature. Water (400 mL) was added to quench the reaction. The solution was extracted with EtOAc (300 mL × 3). The combined organic layer was washed with sat. NaHCO_3_ (350 mL × 1) and brine (350 mL × 2), and dried with anhydrous Na_2_SO_4_, filtered and evaporated under reduced pressure. The residue was purified over a silica gel column (CH_2_Cl_2_–EtOAc, 25:1) to afford **10** (26 g, 56%). mp 136–137 °C;^1^H NMR (400 MHz, DMSO): δ 8.61 (s, 1H), 8.01 (d, 2H, *J* = 8.4 Hz), 7.79 (s, 1H), 7.63 (t, 1H, *J *= 6.8 Hz), 7.55 (d, 2H, *J* = 7.6 Hz), 6.44 (s, 1H), 5.56 (s, 1H), 5.47 (s, 1H), 4.63 (m, 1H), 2.19 (s, 3H), 1.43 (d, 3H, *J* = 6.4 Hz). ^13^C NMR (100 MHz, DMSO): δ 169.1, 165.0, 152.8, 151.2, 150.5, 133.9, 133.4, 132.0, 129.8, 129.0, 128.5, 117.4, 87.8, 81.3, 78.4, 76.8, 76.6, 52.8, 20.6, 14.6; MS (ESI) *m/z* 542.2 [M + H]^+^; HRMS (ESI) *m/z* calcd for [M + H]^+^ C_20_H_18_IClN_3_O_5_: 541.9974, found: 541.9968.

### 3.8. Synthesis of 4-Amino-7-(5′-deoxy-β-D-xylofuranosyl)-5-iodo-pyrrolo*[*2,3-*d]*pyrimidine *1*

A solution of **10** (18.2 g, 33.6 mmol) in methanolic ammonia (saturated with NH_3_ at 0 °C for 2 h, 200 mL) was placed in an autoclave and stirred at 130 °C for 12 h. After cooling, the mixture was concentrated to dryness and the residue was purified over a silica gel column (CH_2_Cl_2_–CH_3_OH, 20:1) to afford **1** (10.3 g, 82%) as yellow powder. mp 190-191 °C; [α]^25^_D_ −68 (*c* 0.1, CH_3_OH); CD: λ_ext_ 240 nm (∆ε −2.6) and λ_ext_ 209 nm (∆ε −4.4);^1^H NMR (400 Hz, DMSO): δ 8.11 (s, 1H, H-2), 7.60 (s, 1H, H-6), 6.73 (brs, 2H, NH_2_), 5.96 (d, 1H, *J *= 1.8 Hz, H-1′), 5.82 (d, 1H, *J* = 3.6 Hz, OH-2′), 5.69 (d, 1H, *J *= 4.3 Hz, OH-3′), 4.21 (m, 1H, H-4′), 4.12 (dd, 1H, *J *= 1.8, 3.6 Hz, H-2′), 3.81(dd, 1H, *J* = 3.6, 4.3 Hz, H-3′), 1.21 (d, 3H, *J *= 6.6 Hz, H-5′); ^13^C NMR (100 Hz, DMSO): δ 157.6 (C-4), 152.3 (C-2), 150.0 (C-8), 128.6 (C-6), 103.4 (C-9), 89.8 (C-1′), 82.4 (C-2′), 78.4 (C-4′), 77.0 (C-3′), 51.8 (C-5), 14.4 (C-5′); HRMS (ESI) *m/z* calcd for [M + H]^+^ C_11_H_14_IN_4_O_3_: 377.0105, found: 377.0098.

## 4. Conclusions

In conclusion, a practical and efficient approach for 10 g scale synthesis of marine nucleoside 4-amino-7-(5′-deoxy-β-D-xylofuranosyl)-5-iodo-pyrrolo[2,3-*d*]pyrimidine **1** was developed on the basis of Vorbrüggen glycosylation. It has the merits of cost efficiency, mild reaction conditions, and easy access to diversity-oriented derivatives. We are currently in the process of applying this approach to other 4-amino-7-(5′-deoxy-β-D-xylofuranosyl)-pyrrolo[2,3-*d*]pyrimidine derivatives and studying their biological activities, such as insecticides, which will be reported in due course. 

## References

[1] Duvall L.R. (1963). Tubercidin. Cancer Chemother. Rep..

[2] Hayashi K., Karnio S., Oono Y., Townsend L.B., Nozaki H. (2009). Toyocamycin specifically inhibits auxin signaling mediated by SCF(TIR1) pathway. Phytochemistry.

[3] Stockwin L.H., Yu S.X., Stotler H., Hollingshead M.G., Newton D.L. (2009). ARC (NSC 188491) has identical activity to Sangivamycin (NSC 65346) including inhibition of both P-TEFb and PKC. BMC Cancer.

[4] Suzuki H., Kim S.H., Tahara M., Okazaki K., Okabe T., Wu R.T., Tanaka N. (1987). Potentiation of cytotoxicity of 1-β-D-arabinofuranosylcytosine for K562 human-leukemic cells by cadeguomycin. Cancer Res..

[5] Jimeno J., Faircloth G., Sousa-Faro J., Scheuer P., Rinehart K. (2004). New marine derived anticancer therapeutics—A journey from the sea to clinical trials. Mar. Drugs.

[6] Seela F., Peng X.H., Budow S. (2007). Advances in the synthesis of 7-deazapurine-pyrrolo[2,3-*d*]pyrimidine-2′-deoxyribonucleosides including D-and L-enantiomers, fluoro derivatives and 2′,3′-dideoxyribonucleosides. Curr. Org. Chem..

[7] Knapp S. (1995). Synthesis of complex nucleoside antibiotics. Chem. Rev..

[8] Isono K. (1988). Nucleoside antibiotics-structure, biological-acitvity and biosynthesis. J. Antibiot..

[9] Li K., Li Q.-L., Ji N.-Y., Liu B., Zhang W., Cao X.-P. (2011). Deoxyuridines from the marine sponge associated actinomycete streptomyces microflavus. Mar. Drugs.

[10] Sagar S., Kaur M., Minneman K.P. (2010). Antiviral lead compounds from marine sponges. Mar. Drugs.

[11] Bhakuni D.S., Rawat D.S. (2005). Bioactive Marine Natural Products.

[12] Margiastuti P., Ogi T., Teruya T., Taira J., Suenaga K., Ueda K. (2008). An unusual iodinated 5′-deoxyxylofuranosyl nucleoside from an *Okinawan ascidian*, *Diplosoma* sp.. Chem. Lett..

[13] Vorbruggen H., Krolikiewicz K., Bennua B. (1981). Nucleoside synthesis. 22. Nucleoside synthesis with trimethylsilyl triflate and perchlorate as catalysis. Chem. Ber. Recl..

[14] Niedballa U., Vorbruggen H. (1976). General synthesis of *N*-glycosides. 6. Mechanism of stannic chloride catalyzed silyl Hibert–Johnson reaction. J. Org. Chem..

[15] Vorbrüggen H., Ruh-Pohlenz C. (2001). Handbook of Nucleoside Synthesis.

[16] Bio M.M., Xu F., Waters M., Williams J.M., Savary K.A., Cowden C.J., Yang C.H., Buck E., Song Z.G.J., Tschaen D.M. (2004). Practical synthesis of a potent hepatitis C virus RNA replication inhibitor. J. Org. Chem..

[17] Dempcy R.O., Skibo E.B. (1991). Regioselective synthesis of imidazo [4,5-*g*]quinazoline quinone nucleosides and quinazoline amino nucleosides. Studies of their xanthine-oxidase and purine nucleoside phosphorylase substrate activity. J. Org. Chem..

[18] Ramasamy K., Imamura N., Robins R.K., Revankar G.R. (1987). A facile synthesis of tubercidin and related 7-deazapurine nucleosides via the stereospecific sodium-salt glycosylation procedure. Tetrahedron Lett..

[19] Kazimierczuk Z., Revankar G.R., Robins R.K. (1984). Total synthesis of certain 2-monosubstituted-tubercidin, 6-mono-substituted-tubercidin and 2,6-disubstituted-tubercidin derivatives-synthesis of tubercidin via the sodium-salt glycosylation procedure. Nucleic Acids Res..

[20] Seela F., Westermann B., Bindig U. (1988). Liquid–liquid and solid–liquid phase-transfer glycosylation of pyrrolo[2,3-*d*]pyrimidines-stereospecific synthesis of 2-deoxy-β-D-ribofuranosides related to 2′-deoxy-7-carbaguanosine. J. Chem. Soc. Perkin Trans. 1.

[21] Seela F., Muth H.P., Bindig U. (1988). Synthesis of 6-substituted 7-carbapurine 2′,3′-dideoxynucleosides-solid-liquid phase-transfer glycosylation of 4-chloropyrrolo[2,3-*d*]pyrimidine and deoxygenation of its 2′-deoxyribofuranoside. Synthesis.

[22] Lupke U., Seela F. (1979). Ribosidation of 7*H*-pyrrolo[2,3-*d*] pyrimidin-4(3*H*)-one at N-3. Chem. Ber. Recl..

[23] Wilcox C.S., Otoski R.M. (1986). Stereoselective preparations of ribofuranosyl chlorides and ribofuranosyl acetates-solvent effects and stereoselectivity in the reaction of ribofuranosyl acetates with trimethylallylsilane. Tetrahedron Lett..

[24] Kane P.D., Mann J. (1984). The preparation and utility of ethyl 2-(5′-*O*-tertbutyldimethylsilyl-2′,3′-*O*-isopropylidene-β-D-ribofuranosyl)propenoate as a key intermediate for C-nucleoside synthesis. J. Chem. Soc. Perkin Trans. 1.

[25] Song Y., Ding H.X., Dou Y.H., Yang R.C., Sun Q., Xiao Q., Ju Y. (2011). Efficient and practical synthesis of 5′-deoxytubercidin and its analogues via vorbruggen glycosylation. Synthesis.

[26] Seela F., Ming X. (2007). 7-Functionalized 7-deazapurine β-D- and β-L-ribonucleosides related to tubercidin and 7-deazainosine: glycosylation of pyrrolo 2,3-d pyrimidines with 1-*O*-acetyl-2,3,5-tri-*O*-benzoyl-β-D or β-L-ribofuranose. Tetrahedron.

[27] Seela F., Peng X.H. (2006). Pyrrolo[2,3-*d*]pyrimidine β-L-nucleosides containing 7-deazaadenine, 2-amino-7-deazaadenine, 7-deazaguanine, 7-deazaisoguanine, and 7-deazaxanthine. Collect. Czech. Chem. Commun..

[28] Seela F., Peng X.H. (2006). 7-Functionalized 7-deazapurine ribonucleosides related to 2-aminoadenosine, guanosine, and xanthosine: Glycosylation of pyrrolo 2,3-d pyrimidines with 1-*O*-acetyl-2,3,5-tri-*O*-benzoyl-D-ribofuranose. J. Org. Chem..

[29] Moravcova J., Capkova J., Stanek J. (1994). One-pot synthesis of 1,2-*O*-isopropylidene-α-D-xylofuranose. Carbohydr. Res..

[30] Hildebrandt B., Nakamura Y., Ogawa S. (1991). Practical synthesis of optically pure 3,4-epoxy-5-methyldihydro-2(3*H*)-furanones from D-xylose by regioselective and stereoselective functionalization. Carbohydr. Res..

[31] Mathe C., Imbach J.L., Gosselin G. (2000). 1,2-Di-*O*-acetyl-5-*O*-benzoyl-3-deoxy-L-erythro-pentofuran ose, a convenient precursor for the stereospecific synthesis of nucleoside analogues with the unnatural β-L-configuration. Carbohydr. Res..

[32] Reigan P., Gbaj A., Stratford I.J., Bryce R.A., Freeman S. (2008). Xanthine oxidase-activated prodrugs of thymidine phosphorylase inhibitors. Eur. J. Med. Chem..

[33] Reigan P., Gbaj A., Chinje E., Stratford I.J., Douglas K.T., Freeman S. (2004). Synthesis enzymatic evaluation of xanthine oxidase-activated prodrugs based on inhibitors of thymidine phosphorylase. Biorg. Med. Chem. Lett..

